# Nebulized dexmedetomidine improves pulmonary shunt and lung mechanics during one-lung ventilation: a randomized clinical controlled trial

**DOI:** 10.7717/peerj.9247

**Published:** 2020-06-05

**Authors:** Bo Xu, Hong Gao, Dan Li, Chunxiao Hu, Jianping Yang

**Affiliations:** 1 The Department of Anesthesiology, The First Affiliated Hospital of Soochow University, Suzhou, Jiangsu, China; 2 The Department of Anesthesiology, The Affiliated Wuxi People‘s Hospital of Nanjing Medical University Wuxi, Jiangsu, China

**Keywords:** Nebulizer, Arterial oxygenation, Intrapulmonary shunt, Lung mechanics, One-lung ventilation, Thoracic surgery, Hypoxic pulmonary vasoconstriction, Anesthesia, Dexmedetomidine

## Abstract

**Background:**

Dexmedetomidine (Dex), a selective a_2_-adrenergic receptor agonist, has been previously reported to attenuate intrapulmonary shunt during one-lung ventilation (OLV) and to alleviate bronchoconstriction. However, the therapeutic effects of nebulized Dex on pulmonary shunt and lung mechanics during OLV have not been evaluated. Here we determine whether nebulized dexmedetomidine improved pulmonary shunt and lung mechanics in patients undergoing elective thoracic surgery in a prospective randomized controlled clinical trial.

**Methods:**

One hundred and twenty-eight patients undergoing elective thoracoscopic surgery were included in this study and randomly divided into four groups: 0.9% saline (Placebo group), 0.5 µg/kg (Dex_0.5_ group), 1 µg/kg (Dex_1_ group) and 2 µg/kg (Dex_2_group) dexmedetomidine. After bronchial intubation, patients received different nebulized doses of dexmedetomidine (0.5 µg/kg, 1 µg/kg and 2 µg/kg) or 0.9% saline placebo during two-lung ventilation(TLV). OLV was initiated 15 min after bronchial intubation. Anesthesia was maintained with intravenous infusion of cisatracurium and propofol. Bispectral Index values were maintained within 40–50 by adjusting the infusion of propofol in all groups. Arterial blood gas samples and central venous blood gas samples were taken as follows: 15 min after bronchial intubation during two-lung ventilation (TLV_15_), after 30 and 60 min of OLV (OLV_30_and OLV_60_, respectively) and 15 min after reinstitution of TLV (ReTLV). Dynamic compliance was also calculated at TLV_15_, OLV_30_, OLV_60_ and ReTLV.

**Results:**

Dex decreased the requirement of propofol in a dose-dependent manner(*P* = 0.000). Heart rate (HR) and mean arterial pressure (MAP) displayed no significant difference among groups (*P* = 0.397 and 0.863). Compared with the placebo group, Dex administered between 0.5 and 2 µg/kg increased partial pressure of oxygen (P_a_O_2_) significantly at OLV_30_ and OLV_60_(*P* = 0.000); however, Dex administered between 1 and 2 µg/kg decreased pulmonary shunt fraction (Q_s_/Q_t_) at OLV_30_ and OLV_60_(*P* = 0.000). Compared with the placebo group, there were significant increases with dynamic compliance (Cdyn) after OLV in Dex_0.5_, Dex_1_ and Dex_2_group(*P* = 0.000). **Conclusions.** Nebulized dexmedetomidine improved oxygenation not only by decreasing pulmonary shunt but also by improving lung compliance during OLV, which may be effective in managing OLV.

## Introduction

Thoracic surgical procedure frequently requires one-lung ventilation (OLV) to improve the operational field of vision and access to the operative space. However, OLV is commonly associated with hypoxemia due to intrapulmonary shunt in the nonventilated collapsed lung. Hypoxemia, defined as a drop in arterial hemoglobin oxygen saturation(S_a_O_2_), therefore leads to acute hypoxic pulmonary vasoconstriction (HPV) (Cheng et al., 2017). HPV is an important protective mechanism by which blood flow is diverted from the nonventilated lung toward a better-ventilated region, thereby maintaining adequate arterial oxygenation. However, many anesthetics, such as inhalation anesthetics and propofol, have shown positive evidence of inhibiting HPV and increasing hypoxemia (Lumb & Slinger, 2015).

Dexmedetomidine (Dex)is an a_2_-adrenoreceptor agonist that has found increasing clinical use—for lung protection, for gentle emergence from anesthesia, as an analgesic, as an adjuvant to local anesthetics during regional anesthesia, and even as a supplemental sedative/anxiolytic (Barends et al., 2017; Nguyen et al., 2017). In the last few years, some studies have shown that nebulized Dex administration may allow minimal systemic effects and rapid drug absorption through the respiratory mucosa (Zanaty & El Metainy, 2015; Abdel-Ghaffar et al., 2018). Although nebulized drug administration may be preferred through pulmonary delivery, there are currently no data describing the nebulized effects of Dex on arterial oxygenation during OLV. This study was designed to test the hypothesis that nebulized Dex may improve arterial oxygenation during OLV. Additionally, this study was meant to explore the feasibility of the application of nebulized Dex in OLV during elective thoracic surgery.

## Materials & Methods

### Study design and participants

**Figure 1:**
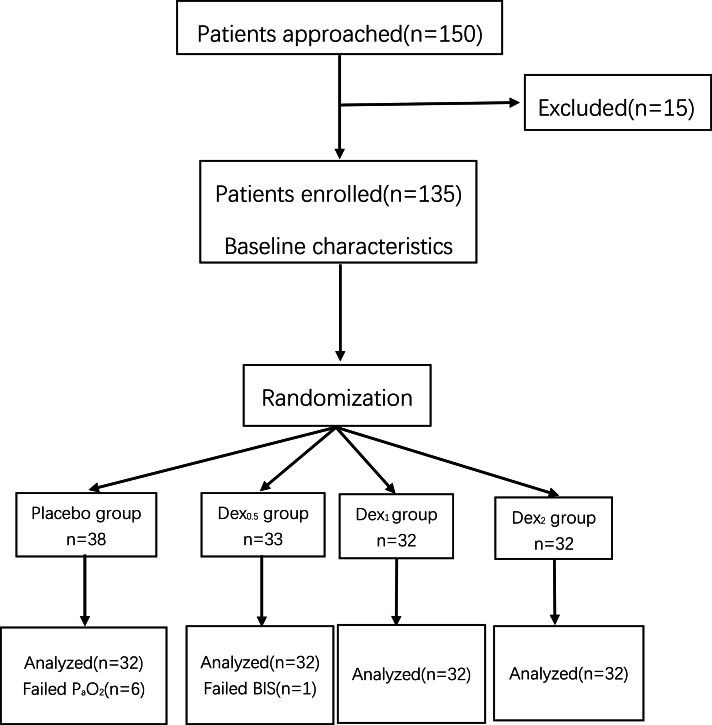
CONSORT flow diagram among four groups. Placebo group, 0.9% saline. Dex_0.5_ group, 0.5 µg/kg Dex. Dex_1_ group, 1 µg/kg Dex. Dex_2_ group, 2 µg/kg Dex.

This study was a prospective, randomized controlled clinical trial, performed in Wuxi People’s Hospital in China. These researcher obtained ethical approval for the study protocol from the Medical Ethics Committee of Wuxi People’s Hospital (Ethical Application Ref: KYuKS201816). This study was registered at the Chinese Ethics Committee of Registering Clinical Trials (ChiCTR1800020112). One hundred and fifty patients undergoing elective thoracoscopic surgery were approached and 128 of them completed the study (Fig. 1). Written informed consent was taken from all participants. The inclusion criteria were as follow: an American Society of Anesthesiologists Physical Status rating of I to II ,aged 20–80 years and height 150–180 cm. Patients with the following conditions were excluded from participation: previous allergic reaction to Dex, serious cardiovascular disorders, liver or kidney dysfunction, arrhythmia, hypertensive patients, severe neuropsychiatric disease, long-term alcohol dependence, or other drug addiction. Patients were randomly allocated into four study groups: 0.9% saline (placebo group), 0.5 µg/kg (Dex_0.5_ group), 1 µg/kg (Dex_1_ group) or 2 µg/kg (Dex_2_ group). Randomization was performed by a computer-generated randomization table, with group allocation concealed in sealed opaque envelopes. An anesthesia nurse who was not involved in the research study was tasked with opening the envelopes 1 h prior to induction of anesthesia and preparation of dexmedetomidine (Jiangsu Hengrui Medicine Co., Ltd) or placebo in identical nebulizer with matching randomization codes. Dexmedetomidine (0.5 µg/kg, 1 µg/kg and 2 µg/kg) was diluted with 0.9% saline into 5 ml and an equal volume of 0.9% saline was used as a control in the placebo group. All preoperative and intraoperative management was performed by the same anesthesiologist who was blinded to the study drug. All the data analysis and statistical evaluation was completed by a professional assistant who was blinded to the patient group allocation.

### Study protocol

**Figure 2:**
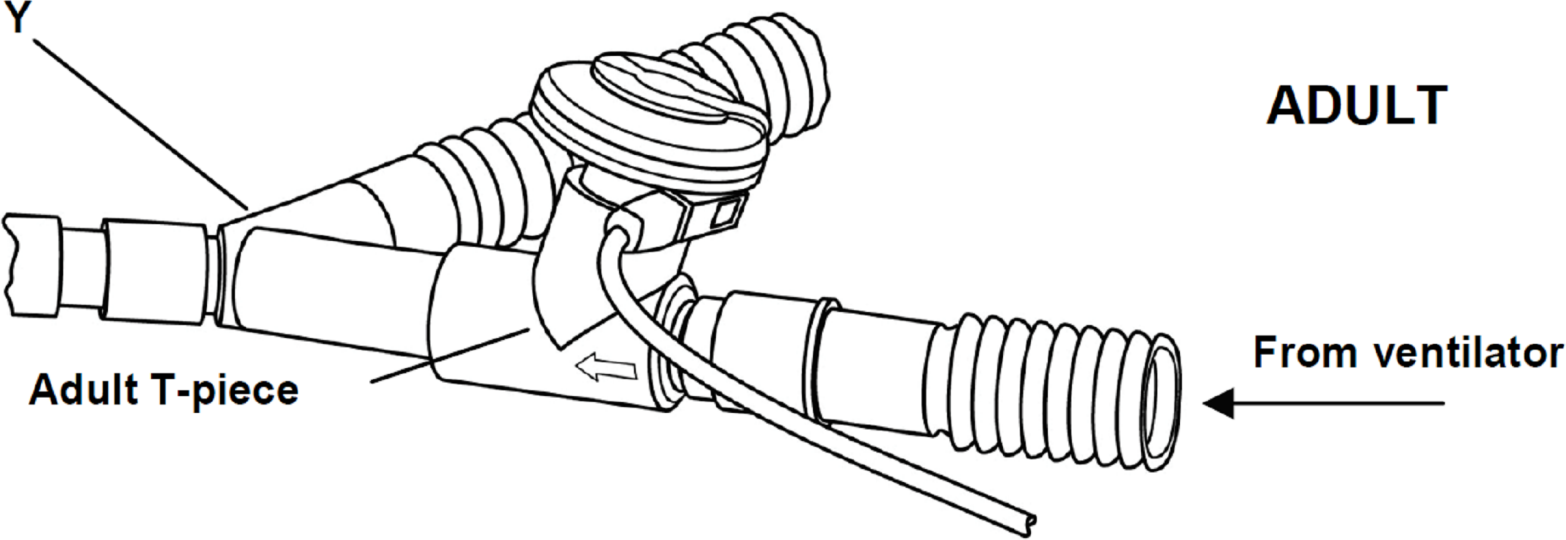
The ventilator circuit. The vibrating mesh nebulizer (Aeroneb Solo, Aerogen, Galway, Ireland) was connected to the circuit with adult T-piece. The devices were placed in the inspiratory limb before the Y-piece.

Patients were monitored by standard monitoring devices upon arrival at the operating room. A 22-gauge arterial catheter (Braun Co.) was inserted in the right or left radial artery. A central venous catheter was placed through the right internal jugular vein and therefore the tip would lie near the right atrium. After anesthesia was induced with midazolam(0.05 mg/kg), sufentanil (0.3–0.4 µg/kg), propofol(1.5–2 mg/kg) and cisatracurium (0.15 mg/kg), bronchial intubation was performed with a left-sided double lumen tube (size 37/35 for males and 35/33 for females), then the correct position was confirmed using fiberoptic bronchoscopy. Mechanical ventilation is used in a pressure-controlled mode for protective ventilation during the study. The ventilator parameters were as follow: inspiratory pressure (P_insp_ ) 20 cm H_2_O, respiratory quotient(I:E) 1:2, oxygen concentration (FO_2_) 100% and respiratory rate adjusted to maintain end-tidal carbon dioxide partial pressure (P_Et_CO _2_) between 30 and 35 mmHg. 5 ml of the study drug was immediately administered via the ventilator circuit (Fig. 2) over 10 min after bronchial intubation during two-lung ventilation. OLV was initiated 15 min after bronchial intubation. During the study, anesthesia was maintained within a bispectral index (BIS) range of 40 to 50 using continuously infused propofol and intermittent administration of cisatracurium. Positive end expiratory pressure (PEEP) to the ventilated lung during OLV was applied in patients who failed to maintain adequate oxygenation (S_p_O_2_ >92%) . Patients requiring PEEP or other recruitment maneuvers for oxygenation were excluded from final analysis. Atropine was administered if heart rate(HR) was less than 50, and ephedrine was required to maintain hemodynamic stability if mean arterial pressure (MAP) had more than 20% decrease from baseline.

### Outcome Measures

Arterial and central venous samples were obtained for blood gas analysis at four time points: 15 min after bronchial intubation during two-lung ventilation (TLV_15_), 30 min and 60min after OLV (OLV_30_ and OLV_60_) and 15 min after reinstitution of TLV (ReTLV). Pulmonary shunt fraction (Q_s_/Q_t_) was calculated using the following formula:

Q_s_/Q _t_= (C_c_O_2_ − C_a_O_2_)/(C_c_O_2_ −C_v_O_2_) ×100%.

Whereby C_a_O_2_ (oxygen content of arterial blood) = (P_a_O_2_ ×0.0031) + (Hb ×1.34 ×S_a_O_2_).

C_v_O_2_ (oxygen content of venous blood) = (P_v_O_2_ ×0.0031) +(Hb ×1.34 ×S_v_O_2_).

C_c_O_2_= ([*F*_i_O_2_ × (PB − _p_H_2_O) − (P_a_CO_2_/RQ)] ×0.0031) + (Hb ×1.34).

PB –Barometric pressure (760 mmHg), _p_H_2_O–47 mmHg,

Hb –Hemoglobin, RQ –Respiratory quotient (0.8).

Dynamic compliance(Cdyn) was obtained from the Primus ventilator at TLV_15_, OLV_30_, OLV_60_, and ReTLV. HR and MAP were recorded at these different times.

### Statistical analysis

Sample size calculation was based on the difference of P_a_O_2_ by 40 mmHg between groups with a power of 90% and two-sided a of 0.05 by repeated measures analysis of variance (Lee et al., 2016). The enumeration data (gender, ASA and operative site) were measured by *χ*^2^ test. Data with normal distributions were presented as mean ±standard deviation. Intergroup comparisons were determined by LSD test. Variables with repeated measures such as PaO_2_, Cdyn, Q_s_/Q _t_, HR, MAP and BIS were analyzed using repeated measures analysis of variance. All statistical analyses were performed with SPSS version 23.0(IBM Inc., Armonk, NY, USA) and *P* < 0.05 was considered statistically significant.

## Results

Here we enrolled and completed studies of total 128 patients during this study. The CONSORT flow diagram is shown in Fig. 1.

Demographic and perioperative data are detailed in Table 1. Overall, there were no differences among groups regarding age (*P* = 0.960), ASA(*P* = 0.982), gender(*P* = 0.919), weight(*P* = 0.204), height(*P* = 0.259), operative site(*P* = 0.988), operational time(*P* = 0.648), crystalloid(*P* = 0.611), colloid(*P* = 0.675), urine(*P* = 0.291), PH(*P* = 0.978), P_a_O_2_ (*P* = 0.547), P_a_CO_2_(*P* = 0.791), Hb(*P* = 0.934), FEV_1_(*P* = 0.705), FVC(*P* = 0.417) and FEV_1∕_FVC(*P* = 0.809).

**Table 1:** Demographic and perioperative data (*N* = 32). All data are expressed as means ± SD.

**Parameters**	**Groups**				**Significance(*P*)**
	**Placebo**	**Dex**_**0.5**_	**Dex**_**1**_	**Dex**_**2**_	
Age(year)	55.5 ± 11.9	55.6 ± 13.0	56.9 ± 8.9	56.3 ± 12.3	0.960
ASA I/II(n)	27/5	26/6	27/5	27/5	0.982
Gender(male/female)	15/17	17/15	17/15	15/17	0.919
Weight(kg)	62.8 ± 11.4	65.0 ± 11.5	60.1 ± 9.6	60.1 ± 10.4	0.204
Height(cm)	164.4 ± 7.6	167.3 ± 7.7	165.1 ± 5.6	164.4 ± 5.2	0.259
Operative site(Right/Left)	14/18	13/19	14/18	13/19	0.988
Operational time(min)	117.9 ± 25.8	116.3 ± 25.9	112.3 ± 21.8	111.8 ± 19.0	0.648
Crystalloid(ml)	571.9 ± 172.7	521.9 ± 131.3	562.5 ± 164.1	553.1 ± 158.6	0.611
Colloid(ml)	440.6 ± 126.6	462.5 ± 100.8	435.9 ± 133.3	425.6 ± 128.4	0.675
Urine(ml)	168.1 ± 38.1	184.1 ± 43.5	168.1 ± 53.5	163.4 ± 45.6	0.291
PH	7.41 ± 0.03	7.41 ± 0.02	7.40 ± 0.03	7.40 ± 0.02	0.978
P_a_O_2_(mmHg)	84.3 ± 5.8	83.6 ± 6.3	82.9 ± 4.8	82.3 ± 6.2	0.547
P_a_CO_2_(mmHg)	35.3 ± 1.5	35.3 ± 1.5	35.2 ± 1.4	35.0 ± 1.0	0.791
Hb(mg l^−1^)	117.2 ± 12.0	117.2 ± 11.7	118.3 ± 12.5	118.8 ± 11.9	0.934
FEV_1_(%)	80.5 ± 10.3	82.0 ± 9.5	79.2 ± 10.4	80.0 ± 8.5	0.705
FVC(% )	88.2 ± 8.5	88.8 ± 7.8	86.1 ± 8.9	86.2 ± 6.9	0.417
FEV_1_/FVC(%)	84.3 ± 8.5	85.6 ± 8.2	83.7 ± 8.7	84.0 ± 7.5	0.809

**Notes.**

Compared with Placebo group.

* *P* < 0.05.

ASA American society of anesthesiologists physical status PH Hydrogen ion concentration P_a_O_2_ Arterial oxygen partial pressure; P_a_CO_2_ Arterial partial pressure of carbon dioxide Hb Hemoglobin concentrations FEV_1_ Forced expiratory volume in one second FVC Forced vital capacity

The values of HR, MAP and BIS weren’t significantly different among groups (*P* = 0.397, 0.863 and 0.815, respectively). During the transition from TLV_15_ to OLV, P_a_O_2_ and Cdyn decreased and Q_s_/Q_t_ increased significantly in the four groups (*P* = 0.000). During OLV, P_a_O_2_ and Cdyn had a significant increase in Dex_0.5_, Dex_1_ and Dex_2_ groups compared with the placebo group (*P* = 0.000) while Q_s_/Q _t_ decreased in the Dex_1_ and Dex_2_ groups compared with the placebo group (*P* = 0.000).

Patients in the Dex_1_ and Dex_2_ group required significantly less propofol during surgery than patients in the placebo group to maintain BIS values between 40 and 50(*P* = 0.000) (Tables 2 and 3). However, the requirement of sufentanil, cisatracurium, ephedrine and atropine did not differ significantly among groups(*P* = 0.728, 0.204, 1.0 and 1.0, respectively) (Table 3).

**Table 2:** Changes of hemodynamics and respiratory mechanics (*N* = 32). All data are expressed as means ± SD.

Parameters	Groups	TLV_1_5min	OLV_3_0min	OLV_6_0min	ReTLV	*P* value
P_a_O_2_(mmHg)						0.000^b^
	Placebo	431.8 ± 54.3	168.6 ± 43.6	178.5 ± 41.3	402.1 ± 42.1	0.000
	Dex_0.5_	435.8 ± 44.1	217.9 ± 43.5^*^	255.6 ± 47.0^*^	418.7 ± 33.2	0.000
	Dex_1_	424.5 ± 38.7	242.5 ± 60.8^*^	282.1 ± 54.6^*^	405.8 ± 37.8	0.000
	Dex_2_	423.4 ± 53.3	262.7 ± 53.6^*^	298.6 ± 38.4^*^	409.1 ± 51.6	0.000
	*P* value	0.691	0.000	0.000	0.429	0.000^a^(*P* = 0.000^c^)
Cdyn (ml/cmH_2_O)						0.000^b^
	Placebo	43.4 ± 7.1	21.0 ± 2.8	19.7 ± 2.8	32.4 ± 2.7	0.000
	Dex_0.5_	42.8 ± 6.0	26.7 ± 2.4^*^	26.2 ± 2.4^*^	38.7 ± 2.6^*^	0.000
	Dex_1_	42.2 ± 5.3	26.4 ± 2.6^*^	25.5 ± 2.6^*^	37.8 ± 1.8^*^	0.000
	Dex_2_	41.5 ± 4.3	26.9 ± 3.2^*^	26.2 ± 2.9^*^	38.5 ± 2.5^*^	0.000
	*P* value	0.619	0.000	0.000	0.000	0.000^a^(*P* = 0.000^c^)
Q_s_/Q_t_ (%)						0.000^b^
	Placebo	9.9 ± 2.2	30.4 ± 2.3	27.5 ± 1.4	9.5 ± 0.5	0.000
	Dex_0.5_	9.8 ± 1.2	30.0 ± 3.0	27.2 ± 2.5	9.4 ± 0.7	0.000
	Dex_1_	9.4 ± 2.0	24.6 ± 2.2^*^	22.3 ± 3.6^*^	9.4 ± 0.6	0.000
	Dex_2_	9.7 ± 2.3	22.6 ± 2.5^*^	21.6 ± 3.1^*^	9.8 ± 0.6	0.000
	*P* value	0.789	0.000	0.000	0.112	0.000^a^(*P* = 0.000^c^)
HR (bpm)						0.000^b^
	Placebo	68.9 ± 9.3	74.6 ± 11.4	76.0 ± 10.3	79.3 ± 10.9	0.002
	Dex_0.5_	70.4 ± 11.9	73.6 ± 13.2	72.8 ± 12.3	74.3 ± 13.1	0.640
	Dex_1_	72.1 ± 10.3	71.6 ± 11.0	71.0 ± 11.5	74.9 ± 9.7	0.478
	Dex_2_	68.3 ± 8.4	73.0 ± 10.0	70.3 ± 8.0	72.6 ± 9.0	0.121
	*P* value	0.424	0.772	0.143	0.086	0.397^a^(*P* = 0.064^c^)
MAP (mmHg)						0.000^b^
	Placebo	82.0 ± 8.2	84.1 ± 9.5	83.0 ± 9.2	92.0 ± 10.2	0.000
	Dex_0.5_	83.3 ± 10.6	81.5 ± 9.8	84.4 ± 8.7	88.3 ± 9.3	0.043
	Dex_1_	85.6 ± 18.1	84.2 ± 8.5	83.6 ± 9.3	90.0 ± 9.1	0.132
	Dex_2_	84.7 ± 6.8	82.2 ± 11.0	83.0 ± 9.3	90.7 ± 10.8	0.002
	*P* value	0.633	0.611	0.914	0.502	0.863^a^(*P* = 0.580^c^)
BIS						0.000^b^
	Placebo	45.6 ± 2.9	43.5 ± 2.2	44.1 ± 2.0	47.5 ± 2.0	0.000
	Dex_0.5_	45.4 ± 2.4	43.2 ± 2.4	43.9 ± 2.3	47.2 ± 1.3	0.000
	Dex_1_	45.6 ± 2.9	43.0 ± 2.4	44.8 ± 2.0	47.6 ± 2.0	0.000
	Dex_2_	45.3 ± 2.8	42.9 ± 2.2	44.9 ± 2.3	47.8 ± 2.5	0.000
	*P* value	0.974	0.727	0.171	0.759	0.815^a^(*P* = 0.650^c^)

**Notes.**

Compared with Placebo group.

* *P* < 0.05.

a *P* value of main effect(group).

b *P* value of main effect(time).

c *P* value of crossover effect.

**Table 3:** Amount of anesthetic and hemodynamic agents administrated during OLV (*N* = 32). All data are expressed as means ± SD.

**Parameters**	**Groups**					**Significance(*P*)**
	**Placebo**	**Dex**_**0.5**_		**Dex**_**1**_	**Dex**_**2**_	
Propofol(mg)	491.3 ± 25.6		494.5 ± 28.3	444.1 ± 20.8^*^	388.3 ± 23.7^*^	0.000
Sufentanil(µg)	48.3 ± 6.4		47.5 ± 6.3	47.0 ± 5.3	46.8 ± 5.2	0.728
Cisatracurium(mg)	24.4 ± 1.7		24.8 ± 1.7	24.0 ± 1.4	24.0 ± 1.6	0.204
Ephedrine(mg)	0.3 ± 1.3		0.3 ± 1.2	0.3 ± 1.2	0.3 ± 1.2	1.0
Atropine(mg)	0.0 ± 0.1		0.0 ± 0.1	0.0 ± 0.1	0.0 ± 0.1	1.0

**Notes.**

Compared with Placebo group.

* *P* < 0.05.

## Discussion

Intraoperative hypoxemia can lead to long term complications including end organ failure and increased mortality. One two-center study of a large non-cardiac surgical population estimated that one in fifteen patients experienced hypoxemia for at least 2 min and that one in sixty-four patients experienced hypoxemia for a minimum of 5 min (Ehrenfeld et al., 2010). The decreased arterial oxygen tension (PaO_2_) may be a reliable indicator of abnormal lung function and predict intraoperative hypoxemia during OLV (Karzai & Schwarzkopf, 2009). Anesthetics may negatively impact the hemodynamic changes that influence PaO_2_. For instance, isoflurane and halothane reduce HPV by up to 50% at 2 MAC and increase the shunt fraction and risk of hypoxia (Saraswat, 2015). Since a majority of intraoperative hypoxemia occurs during OLV, emphasis remains on the discovery and development of appropriate anesthetic agents.

Dex was applied to produce a level of sedation during the perioperative period and in mechanically ventilated patients (Chen & Shen, 2014; Saraswat, 2015). In a recent meta-analysis of 14 randomized controlled trials, Dex was found to increase oxygenation index in seven studies, decrease intrapulmonary shunt in five studies, decrease perioperative HR and MAP in nine studies, and reduce the concentration of the inflammatory factors TNF-α and IL-6 in four studies during OLV (Huang et al., 2017). Intravenous Dex along with isoflurane inhalation was shown to increase P_a_O_2_ and significantly decrease Q_s_/Q_t_ compared to a saline + isoflurane group in a population of adults undergoing elective thoracic surgery (Xia et al., 2013), indicating its safety and feasibility during OLV.

Dex is commonly delivered intravenously but can also be administered as a nebulized premedication in uncooperative children. Inhalation of nebulized Dex is an alternative method of administration, which is related to high bioavailability of Dex (Anttila et al., 2003; McCormick et al., 2008). Nebulized respiratory administration may results in maximizing the surface area of absorption, less drug loss and increased clinical effectiveness (Wolfe & Braude, 2010). In pediatric populations, it has been demonstrated nebulized Dex (as a premedicant) significantly improved cannulating conditions such as parental separation, face mask acceptance and IV placement (Abdel-Ghaffar et al., 2018), with no hemodynamic side-effects (Zanaty & El Metainy, 2015; Abdel-Ghaffar et al., 2018). However, these studies were often applied to pediatric populations.

In the present study, we evaluated whether nebulized Dex could improve oxygenation in adult patients undergoing thoracic surgery. The major finding of this study was that nebulized Dex increased P_a_O_2_ during OLV. This is consistent with the results of previous study done by Kernan et al. (2011) and Xia et al. (2013). We surmise this finding may be associated with the fact that intravenous Dex improved oxygenation and lung mechanics during OLV. Additionally, there was no marked systemic hemodynamic change in our study. This may be due to the tiny systemic effect of nebulized Dex. In contrast, Nguyen et al. (2017) found that intravenous Dex can cause hypotension and bradycardia. Thus, this is especially meaningful as nebulized Dex is superior to standard intravenous route.

We also found that 0.5–2 µg/kg nebulized Dex significantly increased dynamic compliance during OLV. These results were in line with intravenous administration in chronic obstructive pulmonary disease (COPD) patients undergoing lung cancer surgery (Lee et al., 2016). Similar results have been reported in a study of morbidly obese patients with restrictive lung disease undergoing bariatric surgery (Hasanin et al., 2018). However, Groeben has been previously reported that inhalation of Dex caused significant bronchoconstriction in an animal study in contrast to intravenous administration (Groeben, Mitzner & Brown, 2004). This initial bronchoconstriction is because of a variety of factors. Firstly, aerosols of water generated ultrasonically to provoke bronchoconstriction in persons without asthma (Gonzalez et al., 1994; Groeben et al., 2000). Secondly, the initial bronchoconstriction may result from release of inflammatory mediators on airway smooth muscle or activating reflexes mediated via afferent fibers of the vagus nerve (Gonzalez et al., 1994; Bulut, Hirshman & Brown, 1996). Nevertheless, subsequent bronchodilation can be demonstrated after airway was irritated by aerosolized drugs. Thus, it should be likely that Dex may directly improve oxygenation by stimulating bronchodilation to increase dynamic compliance during OLV, though this remains to be substantiated in future studies.

Another finding of this study was that the utilization of nebulized Dex significantly reduced the requirements of anesthetic, specifically propofol. This phenomenon has been previously characterized in intravenous Dex, which decreased the need of isoflurane (Xia et al., 2013) and propofol (Sen et al., 2013) during elective spinal surgery. Although bradycardia and hypotension have been reported in the adult population, patients receiving nebulized Dex did not require more atrophine and ephedrine to maintain adequate hemodynamic stability. Thus it’s likely that nebulized Dex may reduce haemodynamic changes related to propofol (Sherman & Barrick, 2019).

Interestingly, 1–2 µg/kg Dex reduced Qs/Qt and improved oxygenation during OLV. This may be due to a variety of factors. Firstly, the attenuation of local inflammation factors contributing to the hypoxic vasodilator effect of OLV may have been influenced by Dex as has been characterized to diminish the production of these pro-inflammatory factors (Zhang et al., 2018; Meng et al., 2018). Secondly, Dex may have played a direct role in pulmonary artery mechanics, promoting the occurrence of HPV by directly impacting bronchodilation (Lee et al., 2016). Finally, reduction in the requirement of propofol, which has been shown to attenuate HPV in a dose-dependent manner, may have contributed to decreased pulmonary shunt and improved HPV (Huang et al., 2017). All of the above mechanisms are based solely on pathophysiologic speculations and remain to be further ascertained.

Despite the novel findings of the present study, there are several limitations to consider. Firstly, this study used central venous samples instead of mixed venous samples as pulmonary catheter placement isn’t routine in thoracotomy cases at the participating hospital. However, this technique has been validated and used in many earlier studies (Turnaoglu et al., 2001; Ozcan et al., 2007). Secondly, both left and right thoracotomies and multiple lung pathologies were included in the study. In the future, designing a study aimed at patients with shared trauma and sided thoracotomy may bring to light more accurate results. Thirdly, studies with larger sample sizes are warranted to analyze the safety of nebulized Dex.

## Conclusion

Nebulized dexmedetomidine improved oxygenation not only by reducing intrapulmonary shunt but also by increasing lung compliance during OLV. This strategy simultaneously decreased the requirement of propofol without hemodynamic instability. Thus, this study demonstrated that nebulized dexmedetomidine can be used as a feasible strategy for improving oxygenation during OLV in patients undergoing thoracotomy. Nonetheless, studies with larger sample sizes are necessary to clarify the effects and the safety of nebulized Dex used.

##  Supplemental Information

10.7717/peerj.9247/supp-1 Supplemental Information 1 Raw data Demographic and perioperative data; changes of hemodynamics and respiratory mechanics; amount of anesthetic and hemodynamic agents administrated during OLV.

10.7717/peerj.9247/supp-2 Supplemental Information 2 Trial protocol

10.7717/peerj.9247/supp-3 Supplemental Information 3 CONSORT 2010 checklist of information to include when reporting a randomised trial

## Additional Information and Declarations

### Data Availability

The following information was supplied regarding data availability: The raw data are available in Dataset S1.

### Clinical Trial Registration

The following information was supplied regarding Clinical Trial registration: Chinese Clinical Trial Registry:ChiCTR1800020112.
