# Supplementary material for: Nebulized dexmedetomidine improves pulmonary shunt and lung mechanics during one-lung ventilation: a randomized clinical controlled trial
Source: PeerJ. 2020 Jun 5;8:e9247. doi: 10.7717/peerj.9247 (PMC7278887; doi:10.7717/peerj.9247)
Supplement: Supplemental Information 2 [file peerj-08-9247-s002.docx]

**Summary of protocol：**

Thoracic surgery often requires one-lung ventilation (OLV) to improve the operational field of vision and access to the operative space. However, OLV is commonly associated with hypoxemia due to intrapulmonary shunt in the nonventilated collapsed lung. Hypoxemia, defined as a drop in arterial hemoglobin oxygen saturation of <90%, consequently leads to acute hypoxic pulmonary vasoconstriction (HPV). HPV is an important protective mechanism by which blood flow is diverted from the nonventilated lung toward a better‑ventilated region, thereby maintaining adequate arterial oxygenation. However, many anesthetics, such as inhalation anesthetics and propofol, have shown positive evidence of inhibiting HPV and increasing hypoxemia. In the last few years，intravenous injection of dexmedetomidine improve arterial oxygenation during OLV and causes bradycardia. There are currently no data describing the nebulized effects of Dex on arterial oxygenation during OLV. This study was designed to test the hypothesis that nebulized Dex may improve arterial oxygenation during OLV. Additionally, this study was meant to explore the safety and feasibility of the application of nebulized Dex in OLV during elective thoracic surgery.

**Study objective**

To determine whether nebulized dexmedetomidine improved pulmonary shunt and lung mechanics in patients undergoing elective thoracic surgery.

**Study design**

Prospective randomize controlled clinical trial

**Interventions:**

Nebulized inhalation of 0.9% saline or dexmedetomidine(0.5 μg/kg, 1 μg/kg and 2 μg/kg)

**Sample size:**

128

**Outcomes**

P_a_O_2_

Cdyn

Qs/Qt (%)

HR

MAP

BIS

Amount of anesthetic and hemodynamic agents administrated

**Participant selection and enrollment:**

**Subjects will be recruited by:**

Research associate

**Inclusion criteria:**

aged 20 to 80 years, weight 45–85 kg, height 150–180 cm, an American Society of Anesthesiologists Physical Status rating of I to II and undergoing elective thoracoscopic surgery

**Exclusion criteria:**

previous allergic reaction to Dex, serious cardiovascular disorders, liver or kidney dysfunction, arrhythmia, hypertensive patients, severe neuropsychiatric disease, long-term alcohol dependence, or other drug addiction.

**Summary of study procedure:**

Patients were monitored by standard monitoring devices upon arrival at the operating room. After 22-gauge radial arterial catheter was inserted, anesthesia was induced with midazolam 0.05 mg/kg, sufentanil 0.3-0.4 μg/kg, propofol 1.5-2 mg/kg and cisatracurium 0.15 mg/kg. Tracheal intubation was performed with a left-sided double lumen tube (size 37/35 for males and 35/33 for females), and the position of the double-lumen tube was confirmed with a fiber-optic bronchoscope. After induction of anesthesia, the vibrating mesh nebulizer unit was connected with adult T-piece into the inspiratory limb of the breathing circuit before the already anesthetized patient. After adult breathing circuits were installed for use, 5 ml of the allocated solution was administered via the nebulizer as an aerosol over 10 min during two-lung ventilation, with pressure-control ventilation: P_insp_ = 20cmH_2_O, I：E = 1:2, F_i_O_2_ = 100 % and adjusted RR according to P_Et_CO_2_ (30-35 mmHg). A central venous catheter was placed into the right atrium through the right internal jugular vein, 15–18 cm deep, equivalent to the level of the right atrium. After positioning the patient in the lateral decubitus position, OLV was initiated. Anesthesia depth was maintained at Bispectral Index 40-50 by continuous intravenous infusion of propofol and intermittent administration of cisatracurium. Atropine and ephedrine were used when required to maintain hemodynamic stability. Positive end expiratory pressure (PEEP) to the dependent lung was applied in patients who failed to maintain adequate oxygenation (S_p_O_2_ >92%) with the aforementioned ventilator settings. Patients requiring PEEP or other recruitment maneuvers for oxygenation were excluded from final analysis.

Outcome Measures

Arterial and central venous samples were obtained for blood gas analysis at four time points: 15 min after intubation during two-lung ventilation (TLV_15_), after 30 min, and 60min of OLV (OLV_30_, and OLV_60_) and 15 min after reinstitution of TLV (ReTLV). Pulmonary shunt fraction (Q_s_/Q_t_) was calculated using the following formula:

Q_s_/Q_t_ = (C_c_O_2_ − C_a_O_2_)/(C_c_O_2_ − C_v_O_2_) x 100 %.

Whereby C_a_O_2_ (oxygen content of arterial blood) = (P_a_O_2_ × 0.0031) + (Hb × 1.34 × S_a_O_2_).

C_v_O_2_ (oxygen content of venous blood) = (P_v_O_2_ × 0.0031) +(Hb × 1.34 × S_v_O_2_). C_c_O_2_= ([F_i_O_2_ × (PB − _p_H_2_O) − (P_a_CO_2_/RQ)] × 0.0031) + (Hb × 1.34).

PB – Barometric pressure (760 mmHg), _p_H_2_O – 47 mmHg,

Hb – Hemoglobin, RQ – Respiratory quotient (0.8).

Dynamic compliance(Cdyn) was obtained from the Primus ventilator. Heart rate(HR) and mean arterial pressure(MAP) were recorded at TLV_15_, OLV_30_, OLV_60_, and ReTLV.

**Participant confidentiality:**

All data collection instruments are kept in in a secured manner and password protection of all electronic data files will be performed. The hard copies of the data collection forms will be kept in a secured manner in the office of the PI in the Department of anesthesia. Access to these documents will be restricted to study personnel. Data will be deidentified after completion of the study.

**Anticipated risks or adverse events:**

Bradycardia, hyoxemia

**Adverse events reporting:**

The principal investigators will be primarily responsible for monitoring the safety and efficacy of the study, executing the data safety monitoring plan, and complying with all reporting requirements to the ethics committee. The principal investigators will review the approved protocol prior to enrolling research participants and perform regular review of study progress and patient outcomes on a weekly basis.
